# Do Adults Show a Curse of Knowledge in False-Belief Reasoning? A Robust Estimate of the True Effect Size

**DOI:** 10.1371/journal.pone.0092406

**Published:** 2014-03-25

**Authors:** Rachel A. Ryskin, Sarah Brown-Schmidt

**Affiliations:** 1 Department of Psychology, University of Illinois at Urbana-Champaign, Champaign-Urbana, Illinois, United States of America; 2 Beckman Institute for Advanced Science and Technology, Champaign-Urbana, Illinois, United States of America; Birkbeck, University of London, United Kingdom

## Abstract

Seven experiments use large sample sizes to robustly estimate the effect size of a previous finding that adults are more likely to commit egocentric errors in a false-belief task when the egocentric response is plausible in light of their prior knowledge. We estimate the true effect size to be less than half of that reported in the original findings. Even though we found effects in the same direction as the original, they were substantively smaller; the original study would have had less than 33% power to detect an effect of this magnitude. The influence of plausibility on the curse of knowledge in adults appears to be small enough that its impact on real-life perspective-taking may need to be reevaluated.

## Introduction

The ability to represent the beliefs of others is an essential, but non-trivial task. Extensive research demonstrates that young children struggle to separate their private knowledge from the beliefs of others, as measured by tasks that ask the child to reason about the actions of someone who holds a false-belief about, for example, the location of a desired toy [1], [2]. Birch and Bloom [3] compare the child’s difficulty in false-belief tasks with the “Curse of Knowledge,” a phenomenon in which adults are biased towards their own knowledge when attempting to evaluate the views of a more naïve individual [4], [5]. In the false-belief task, difficulty similarly comes from an inability to set aside one’s own egocentric knowledge of reality when reasoning about another’s false beliefs.

In an influential study (159 citations in Google Scholar, 75 in Web of Science), Birch and Bloom [6] examined adults’ susceptibility to the curse of knowledge in reasoning about false-belief. In particular, they tested the hypothesis that adults’ success in a false-belief task would be modulated by the *plausibility* that the other person would act in a manner consistent with their own egocentric knowledge of reality.

**Figure 1:**
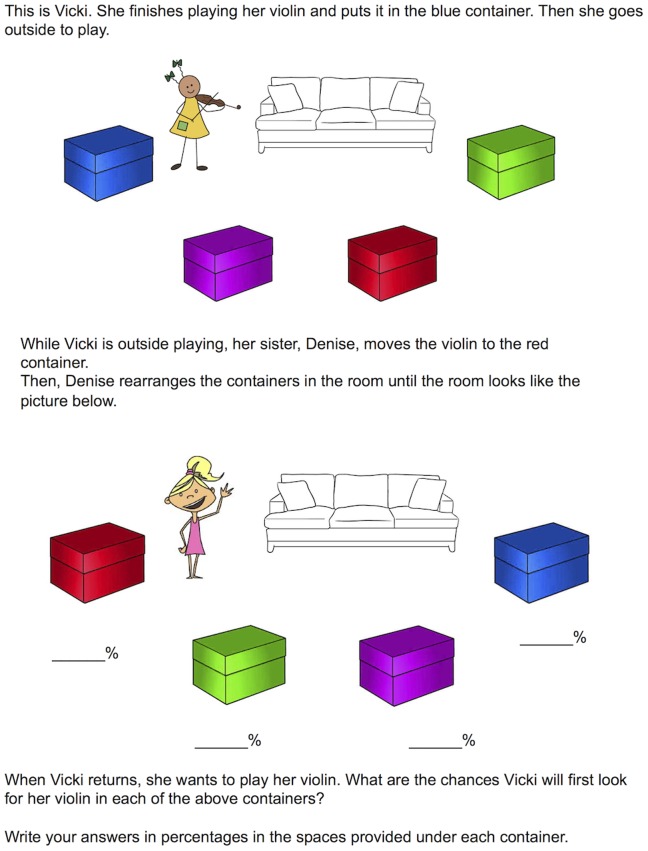
Example vignette. This vignette is nearly identical to the one used in Experiment 2 (the image of Vicki was swapped out for the purposes of this manuscript due to a copyright on the original image).

To test this hypothesis, Birch and Bloom [6] presented young adult participants with a vignette in which Vicki, a girl playing her violin, is pictured in a room with four different-colored containers (Figure 1, also see descriptions of all stimuli in Table 1). She places her violin in the blue container and leaves the room. Her sister Denise then enters and moves the violin to another container–red, purple, or unknown, depending on the condition. The participants then report the probability with which Vicki will look in any one of the containers first.

**Table 1:** Mean ratings of the likelihood that Vicki will look in the red container first by experiment and by condition.

*Ratings of Red container*	Birch & Bloom (2007)		Experiment 1			Experiment 2		Experiment 3		Experiment 4			Experiment 5			Experiment 6			Experiment 7		
	*M(SD)*	*n*		*M (SD)*	*n*	*M (SD)*	*n*	*M (SD)*	*n*		*M (SD)*	*n*		*M (SD)*	*n*		*M (SD)*	*n*		*M (SD)*	*n*
Control	NA		NA			28.63 (25.14)*	134	NA		NA			NA			NA			NA		
Ignorance	23 (22)	56		23.18 (22.10)	65	22.15 (22.86)	132	25.18 (19.25)	107		22.34 (23.80)	103		20.98 (22.37)	104		28.18 (23.51)	73		16.53 (18.28)	309
Knowledge Implausible	19 (21)	43		19.71 (20.60)	198	30.69 (24.97)*	134	22.98 (23.63)	107		16.91 (23.17)	101		22.29 (22.56)	100		22.05 (21.47)	88		16.26 (18.9)	308
Knowledge Plausible	34 (25)*	51		22.75 (23.97)	197	33.35 (25.25)*	127	28.89 (24.11)	105		24.27 (24.27)	100		26.95 (21.59)^ (^*^)^	101		30.86 (25.25)	81		22.08 (21.86)*	300
Display	See Birch and Bloom (2007)		Same as Birch and Bloom (2007) except: a. Different images of Vicki and Denise b. Vicki and Denise are centered c. No couch d.Boxes are closed			Same as Birch and Bloom (2007) except: a. Different images of Vicki and Denise b. Different couch image c. Boxes are closed		Same as Birch and Bloom (2007)		Same as Birch and Bloom (2007)			Same as Birch and Bloom (2007) except: Denise is close to the red box			Same as Birch and Bloom (2007)			Same as Birch and Bloom (2007) except: Counterbalanced locations of the boxes^1^		

Asterisks mark means that differ significantly from those in the *Ignorance* condition of the same experiment (asterisk in parenthesis indicates *p* = 0.05).

1 Order 1 was identical to Birch and Bloom. In order 2, the top part of the vignette was arranged in the following way: red, blue, green, purple. The bottom part was purple, red, blue, green. In order 3, the top part of the vignette was arranged in the following way: purple, green, blue, red. The bottom part was green, blue, red, purple. In order 4, the top part of the vignette was arranged in the following way: green, red, purple, blue. The bottom part was blue, purple, green, red.

**Figure 2:**
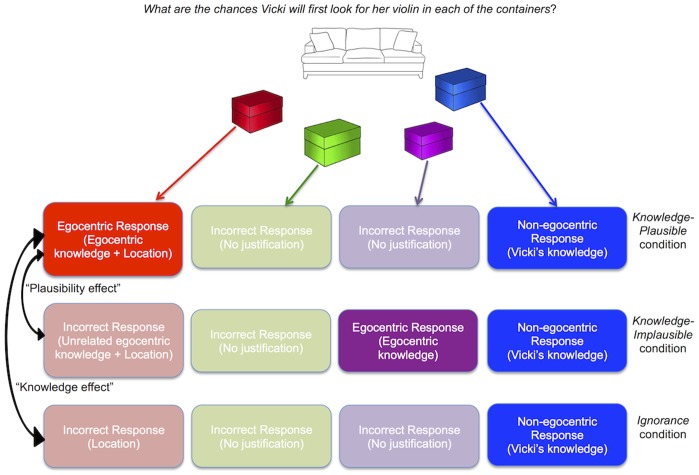
Interpretation of the possible responses in each condition based on the logic of the original experiment. High-ratings of the blue container reflect taking into account Vicki’s knowledge exclusively: a non-egocentric response in all conditions. Inflated ratings of the red container are hypothesized to be driven by its location (previously occupied by the blue container) and, in the *Knowledge-Plausible* condition, egocentric knowledge of the violin’s true location. Therefore, the difference in red-container ratings between *Ignorance* and *Knowledge-Plausible* conditions constitutes an effect of “knowledge.” The difference in red-container ratings between *Knowledge-Implausible* and *Knowledge-Plausible* conditions constitutes an effect of “plausibility,” because, while egocentric knowledge is present in both conditions, it converges on the plausible red-container in the *Plausible* condition only.

The plausibility of Vicki’s actions was manipulated by having Denise rearrange the containers before Vicki’s return, such that the red container is shown where the blue container had been. In the *Knowledge-Plausible* condition, Denise moves the violin to the red container, a plausible search location because that container is located where Vicki had originally hidden her violin (Table 2). In the *Knowledge-Implausible* condition, Denise moves the violin to the purple container, which is in a different spot from where Vicki had originally hidden her violin. To test for a curse of knowledge, performance in the two *Knowledge* conditions is compared to an *Ignorance* condition in which participants do not know which container (red, purple, or green) the violin was moved to (Fig. 2). Birch and Bloom predicted that when egocentric knowledge was consistent with a plausible course of actions (*Knowledge-Plausible* condition), adults might show susceptibility to the curse of knowledge in reasoning about false-belief (i.e., about Vicki’s inaccurate representation of the violin’s location). Further, they claimed that when the egocentric knowledge pointed to a less plausible (*Knowledge-Implausible* condition) place to look – the purple container – there would be no such curse of knowledge. Previous work had suggested that plausibility mediates the magnitude of the curse of knowledge [7], [8].

**Table 2:** Summary of Birch and Bloom (2007) experimental design.

	Red container	Green container	Purple container	Blue container
*Knowledge-Plausible*	**current location of violin**	irrelevant	irrelevant	**original location of violin**
*Knowledge-Implausible*	irrelevant	irrelevant	**current location of violin**	**original location of violin**
*Ignorance*	unknown	unknown	unknown	**original location of violin**

Note from Vicki’s perspective, the violin is always most likely to be in the blue container.

With a sample of about 50 people per condition, Birch and Bloom [6] found evidence for a curse of knowledge modulated by plausibility. In the *Knowledge-Plausible* condition, ratings for the red container (34%) were significantly higher than the *Ignorance* condition (23%), consistent with an inability to ignore egocentric knowledge. Likewise, ratings of the blue container (where *Vicki* believes her violin to be) were lower in the *Knowledge-Plausible* condition (59%) vs. *Ignorance* (71%). Critically, in the *Knowledge-Implausible* condition, ratings of the true location of the violin (purple), and of the belief-container (blue) were not significantly different from the *Ignorance* condition. However, the test of purple-container responses is somewhat inconclusive due to a possible floor effect. While red-container ratings in the *Knowledge-Implausible* (19%) condition were numerically lower than in both the *Ignorance* and *Knowledge-Plausible* conditions, Birch and Bloom did not explicitly report these comparisons.

Birch and Bloom draw two conclusions from these findings: First, egocentric knowledge compromises adults’ ability to reason about another person’s false beliefs. Second, plausibility determines whether adults will suffer from the curse of knowledge; simply having private knowledge that is not shared by Vicki is not enough to elicit the effect. These findings are of particular interest because they suggest that adults’ ability to reason about mental states is fragile and child-like in some circumstances. Further, these findings back claims that adults are inherently egocentric (e.g., [9], [10]).

Here we propose that the comparison of red-container ratings in the *Knowledge-Plausible* and *Knowledge-Implausible* conditions is a crucial step in evaluating whether or not plausibility underlies the curse of knowledge effect. If plausibility has a causal role, as Birch and Bloom claim, the *Knowledge-Plausible* condition should elicit substantially higher ratings of the red container than the *Knowledge-Implausible* condition, which in their experiment it does.

In a series of seven experiments, we find that Birch and Bloom’s original study overestimated the size of the effects of both egocentric knowledge and, in particular, plausibility. By our estimate, the effects are small enough that, in real world situations, they would exert relatively little pressure on perspective-taking behavior.

## Experiments 1–7

The original goal of the present research was to replicate and extend Birch and Bloom’s [6] finding as a metric of theory-of-mind reasoning abilities in adulthood. The results of our efforts, described below, are a series of seven well-powered experiments in which we examine this phenomenon.

Estimates of the effect size were conducted based on Birch and Bloom’s [6] reported means and standard deviations. For the 11% difference in ratings of the red container between *Knowledge-Plausible* and *Ignorance* conditions, the estimated effect size was *d* = .469. Surprisingly, this is only slightly smaller than analogous effects found in three-and four-year-olds, approximately *d* = 0.59 and *d* = 0.55, respectively [3]. For the 15% difference in red-container ratings between *Knowledge-Plausible* and *Knowledge-Implausible* conditions, the estimated effect size was *d* = .645. A power analysis (G*Power 3.1; [11]) indicated that to detect the egocentric knowledge effect (*d* = .469) and achieve 80% power (two-tailed) would require 73 participants per condition (97 for 90% power). Detecting the plausibility effect (*d* = .645) with 80% power would require 39 participants per condition (52 for 90% power).

### Methods

#### Participants

In Experiments 1, 2, 3, and 6, participants were undergraduates at the University of Illinois, Urbana-Champaign. They received partial course credit for participating. In Experiments 4, 5, and 7, the participants were Amazon Mechanical Turk workers from the United States, who received either $0.10 (experiments 4 and 5) or $0.25 (experiment 7) for participating. Previous work shows that the Mechanical Turk population provides reliable data and benefits from more diversity than typical undergraduate samples [12], [13], [14]. The task (in six of the experiments) was carried out online, and took less than 3 minutes to complete. Experiment 6 was conducted at the beginning of several basic psychology classes at the University of Illinois.

### Ethics Statement

The University of Illinois, Urbana-Champaign Institutional Review Board approved this study. Informed written consent was obtained from all participants prior to participation.

### Procedure

Participants first saw a vignette modeled after, or identical to, that used by Birch and Bloom [6]; see Table 1 for exact display descriptions. After completing the vignette, participants answered demographic questions (e.g., age, number of languages spoken, nationality). In the online format, the false-belief task responses were typed in four answer boxes labeled with the corresponding color (from top to bottom: red, green, purple, blue). In Experiment 1, the third container was yellow rather than purple. On paper, the responses were written under each box, as in Birch and Bloom [6]. The online survey only continued if the answers added up to 100%. Participants whose paper responses didn’t add up to 100% were excluded (Expt 2: n = 8; Expt 6: n = 24). Participants were randomly assigned to one of the three conditions (*Knowledge-Plausible, Knowledge-Implausible*, or *Ignorance*) used by Birch and Bloom [6]. The only difference between conditions was the first sentence of the middle portion of text (see Fig. 1 for full text):

*Knowledge-Plausible:* “moves the violin to the red container.”

*Knowledge-Implausible*: “moves the violin to the purple container.”

*Ignorance*: “moves the violin to another container.”

Exp.1 contained an additional short task intended to prime either egocentric or allocentric thinking [15], [16]. The priming conditions were completely crossed with the false-belief conditions. Preliminary analyses showed that priming had no effect on responding (*p*’s>.8) and will not be discussed further. Experiment 2 contained a fourth condition modeled after an experiment by Converse, Lin, Keysar, & Epley [10]. In this control condition, after Vicki leaves the room, Denise enters and rearranges the containers but no mention of the violin is made. A subset of participants in Experiment 2 (n = 152) responded on paper, at the beginning of a psychology class. The pattern of results did not differ based on response medium so we collapsed across these in the analyses. Experiments 3, 4, 6, and 7 used the exact same stimuli as Birch and Bloom [6]. Experiment 7 used a balanced Latin square design to counterbalance the order of the containers (see Table 1). Note, in the original study, the red container was always the first to be rated.

## Results

Following Birch and Bloom [6], the primary measure of susceptibility to the curse of knowledge consists of the ratings of the red container in the *Knowledge-Plausible* compared to the *Ignorance* condition (Means are summarized in Table 1 and inferential statistics are shown in Tables 3 and 4). We additionally conducted planned comparisons of the red-container ratings in the *Knowledge-Plausible* vs. *Knowledge-Implausible* conditions to pinpoint the plausibility of egocentric knowledge as the determinant of the curse of knowledge (rather than the simple fact of holding private knowledge unrelated to the red container, see Fig. 2). The analysis of purple-container ratings was not significant in the original report, thus those comparisons were not part of our planned comparisons. Similarly, we focus on comparisons of red-container ratings because blue-container ratings are non-independent.

**Table 3:** Welch’s two-sample t-tests for ratings of the red container.

	*Knowledge-Plausible vs. Ignorance*	*Knowledge-Plausible vs. Knowledge-Implausible*	*Knowledge-Implausible vs. Ignorance*
*Birch & Bloom*	***t*** **(105) = −2.42, p<.05**	not reported	not reported
*Exp. 1*	*t*(118) = −0.13, p = .89	*t*(384) = 1.35, p = .18	*t*(103) = −1.12, p = .27
*Exp. 2*	***t*** **(252) = 3.74, p<.001**	*t*(258) = 0.85, p = .39	***t*** **(263) = 2.91, p<.005**
*Exp. 3*	*t*(199) = 1.24, p = .22	*t*(210) = 1.80, p = .07	*t*(204) = −0.75, p = .46
*Exp. 4*	*t*(201) = .57, p = .57	***t*** **(198) = 2.20, p<.05**	*t*(202) = −1.65, p = .10
*Exp. 5*	***t*** **(203) = 1.94, p = .053**	*t*(198) = 1.50, p = .13	*t*(202) = .42, p = .68
*Exp. 6*	*t*(152) = .68, p = .50	***t*** **(158) = 2.43, p<.05**	*t*(148) = −1.71, p = .09
*Exp. 7*	***t*** **(582) = 3.39, p<.001**	***t*** **(589) = 3.51, p<.001**	*t*(615) = −.18, p = .85
*All*	***t*** **(1902) = 4.57, p<.001**	***t*** **(2025) = 5.06, p<.001**	*t*(1902) = −0.41, p = .68

The bottom row shows the post-hoc analysis of the combined data from all seven experiments.

**Table 4:** Welch’s two-sample t-tests for ratings of the blue container.

	*Knowledge-Plausible vs. Ignorance*	*Knowledge-Plausible vs. Knowledge-Implausible*	*Knowledge-Implausible vs. Ignorance*
*Birch & Bloom*	***t*** **(105) = 2.35, p<.05**	not reported	*t*(97) = −0.21, n.s.
*Exp. 1*	*t*(115) = −0.03, p = .98	*t*(393) = −0.21, p = .83	*t*(114) = 0.12, p = .90
*Exp. 2*	***t*** **(257) = −2.69, p<.01**	*t*(259) = −0.56, p = .58	***t*** **(263) = −2.16, p<.05**
*Exp. 3*	*t*(207) = −0.27, p = .79	*t*(210) = −0.93, p = .35	*t*(210) = 0.71, p = .48
*Exp. 4*	*t*(200) = .84, p = .40	*t*(199) = −0.80, p = .42	*t*(201) = 1.60, p = .11
*Exp. 5*	*t*(202) = −0.46, p = .65	*t*(195) = −1.14, p = .25	*t*(199) = −1.61, p = .11
*Exp. 6*	*t*(148) = .68, p = .50	*t*(167) = −1.50, p = .14	***t*** **(153) = 2.09, p<.05**
*Exp. 7*	*t*(602) = −0.49, p = .62	*t*(606) = −1.18, p = .24	*t*(612) = .63, p = .53
*All*	*t*(1844) = −1.37, p = .17	***t*** **(2045) = −2.21, p<.05**	*t*(1870) = 0.72, p = .47

The bottom row shows the post-hoc analysis of the combined data from all seven experiments.

### Experiment 1

**Figure 3:**
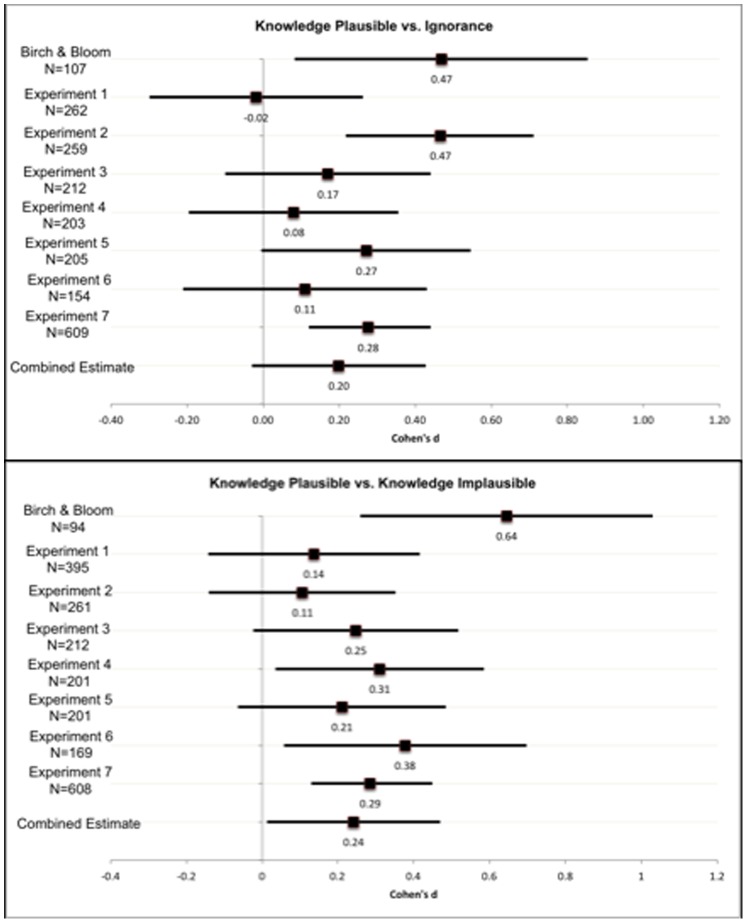
Effect sizes by experiment. Cohen’s *d* (and 95% confidence intervals) of *Knowledge-Plausible* vs. *Ignorance* (top panel), and *Knowledge-Plausible vs. Knowledge-Implausible* (bottom panel) across experiments.

The effect of knowledge (Fig. 3a) was not significant (*t*(118)* = *−0.13, *p* = .89), and was in the opposite direction of Birch and Bloom’s findings. There was no effect of plausibility (Fig. 3b, *t*(384) = 1.35, *p* = .18). These findings do not support the hypothesis that plausible, egocentric knowledge leads to more errors in adults’ false-belief reasoning. To explore the cause of this failure to replicate, Experiments 2–7 are aimed at estimating the true size of this effect.

### Experiment 2

Participants in the *Knowledge-Plausible* condition gave higher ratings to the red container than participants in the *Ignorance* condition (knowledge effect, Fig. 3a), similar to Birch and Bloom’s findings [6]. However, participants in the *Knowledge-Implausible* condition also gave higher ratings to the red than those in the *Ignorance* condition (*d* = .36, Table 1). The effect of plausibility (*Knowledge-Plausible vs. Knowledge-Implausible*) is smaller than the original estimate (Fig. 3b). The *Control* condition did not differ significantly from the *Knowledge-Plausible* condition, *t*(258) = 1.51, *p* = 0.13, inconsistent with the findings of Converse, et al. [10] (using a mood manipulation, they found that this effect did not replicate when participants were sad). Participants in the *Control* condition also showed higher red-container ratings compared to *Ignorance*. The high red-container ratings in the *Knowledge-Implausible* and *Control* conditions suggest that it was not knowledge of the violin’s location in the red container that inflated red-container ratings in the *Knowledge-Plausible* condition.

### Experiment 3

Using twice as many participants as Birch and Bloom [6], the effects of private knowledge and plausibility (Fig. 3a–b) were much smaller than originally estimated, with 95% confidence intervals including zero.

### Experiment 4

The data patterns resemble those of Birch and Bloom, however both the effects of private knowledge and plausibility were much smaller than in the original study (Fig. 3a–b).

### Experiment 5

Ratings of the red container were higher in the *Knowledge-Plausible* than both the *Ignorance* and the *Knowledge-Implausible* conditions, but these effects were much smaller than in Birch and Bloom’s study (Fig. 3a–b).

### Experiment 6

Ratings of the red container were higher in the *Knowledge-Plausible* condition than both the *Ignorance* and the *Knowledge-Implausible* conditions, but these effects were much smaller than in Birch and Bloom’s study (Fig. 3a–b). Additionally, the red-container ratings were much lower in the *Knowledge-Implausible* condition than the *Ignorance* condition; correspondingly, blue box ratings were higher in the *Knowledge-Implausible* condition than the *Ignorance* condition (see Table S1). This data pattern is somewhat challenging to interpret because it suggests that having private knowledge unrelated to the red container makes it *easier* to appreciate Vicki’s belief state.

### Experiment 7

Collapsing across four counterbalanced orders, ratings of the red container were higher in the *Knowledge-Plausible* condition than both the *Ignorance* and the *Knowledge-Implausible* conditions, but these effects were much smaller than in Birch and Bloom’s study (Fig. 3a–b).

### Meta-analysis of the Effect Size

Combining the effect sizes across the seven studies, we estimate the magnitude of the knowledge effect to be *d* = 0.20, less than one-half the original estimate (*d* = .469). The overall plausibility effect was estimated at *d* = 0.24, less than one-third the original estimate (*d* = .645). While these findings suggest the effects of knowledge and plausibility to be real but small, we also point out that in two of the experiments (Experiments 2 and 5), participants gave numerically higher ratings to the red-container when they knew the violin was in the purple container (*Knowledge-Implausible*), compared to the *Ignorance* condition, and the reverse pattern was observed in Experiment 6. This calls into question the validity of the knowledge effect as a test of egocentrism. Instead, the difference in red box ratings between these conditions may reflect multiple, competing variables such as interference due to active maintenance of multiple representations (Vicki’s knowledge state and the conflicting egocentric knowledge), and demand characteristics which may be brought about by situations in which participants are made aware of competing perspectives (e.g., closer attention to Vicky’s knowledge in situations where an alternative perspective is made available, such as the *Knowledge-Implausible* condition).

## Conclusions

Birch and Bloom [6] argued that egocentric knowledge interferes with adults’ ability to reason about the mental states of others. Their findings provided key support for claims that adults lack the ability to use their knowledge about other people’s knowledge and beliefs even when they need it most [10], [17]. Yet, across seven experiments with large samples (total n = 3074), we find that these effects are much smaller than originally estimated, enough so that they may not be key factors in real-life reasoning about the perspectives of others.

Do the results of these attempted replications support the original claims made by Birch and Bloom given that they are in the same direction as the original findings? Following the logic of Simonsohn [18] we consider what effect size the *original* experiment could have detected as one way to judge whether our results should “count” as a replication. If the original study could not reliably detect an effect of the size we found with at least 33% power, we can argue that our result is substantively different from the original. With approximately 50 participants per condition, the original study would have 33% power to detect an effect size of *d = *.31. It would have had approximately 17% and 22% power to detect our effect sizes of *d = .*20 and *d = *.24 respectively. Thus, by this metric, our findings constitute a failure to replicate the original result.

A potentially more important question, however, is whether an effect size of.20 is meaningful? To detect an effect of *d* = .20 with 80% power, a study attempting to replicate this result would require 394 participants *per condition*. These effects are so small as to be impractical to replicate and extend with typically-sized samples. Further, the task is not sensitive or reliable enough to be used as a metric of theory of mind reasoning in individual adults, as we had originally planned to do at the outset of these replication attempts.

At a minimum, the small effect sizes suggest that the effects of private knowledge and plausibility might not be relevant to real-life behavior and reasoning. After all, analyses of moment-by-moment cognitive processes show that even when egocentric biases are detectable, adults rapidly make use of information about the beliefs of others [19], particularly in interactive conversation [20]. Situated within the broader literature on the role of belief-information in cognitive processes, our findings show that this plausibility-modulated curse of knowledge effect should be reevaluated as a key determinant of our ability to gain insights into the minds of others.

## Supporting Information

Table S1 Means and standard deviations of ratings for each container, by condition (all experiments).
(DOCX)
