# Supplementary material for: Do Adults Show a Curse of Knowledge in False-Belief Reasoning? A Robust Estimate of the True Effect Size
Source: PLoS One. 2014 Mar 25;9(3):e92406. doi: 10.1371/journal.pone.0092406 (PMC3965426; doi:10.1371/journal.pone.0092406)
Supplement: Table S1 — Means and standard deviations of ratings for each container, by condition (all experiments). (DOCX) [file pone.0092406.s001.docx]

|  | **Red** | **Green** | **Purple** | **Blue** |
| --- | --- | --- | --- | --- |
|  | *M (SD)* | *M (SD)* | *M (SD)* | *M (SD)* |
| **Experiment 1** |  |  |  |  |
| Ignorance | 23.18 (22.1) | 5.51 (9.44) | 5.45 (9.42) | 65.86 (30.38) |
| Knowledge-Implausible | 19.71 (20.6) | 7.19 (14.53) | 6.69 (13.26) | 66.41 (31.89) |
| Knowledge-Plausible | 22.75 (23.97) | 5.69 (11.8) | 5.83 (11.86) | 65.74 (32.14) |
| **Experiment 2** |  |  |  |  |
| Control | 28.63 (25.14) | 3.84 (7.68) | 4.51 (11.14) | 63.02 (29.66) |
| Ignorance | 22.15 (22.86) | 5.79 (10.02) | 5.01 (8.48) | 67.05 (30.6) |
| Knowledge-Implausible | 30.69 (24.97) | 4.09 (8.54) | 6.11 (15.19) | 59.1 (29.29) |
| Knowledge-Plausible | 33.35 (25.25) | 4.02 (7.88) | 5.55 (13.12) | 57.09 (28.96) |
| **Experiment 3** |  |  |  |  |
| Ignorance | 25.18 (19.25) | 7.11 (10.64) | 6.19 (8.72) | 61.52 (27.61) |
| Knowledge-Implausible | 22.98 (23.63) | 5.83 (10.86) | 6.83 (10.77) | 64.36 (30.41) |
| Knowledge-Plausible | 28.89 (24.11) | 5.36 (10.43) | 5.3 (12.66) | 60.46 (30.51) |
| **Experiment 4** |  |  |  |  |
| Ignorance | 22.34 (23.8) | 6.8 (10.93) | 7.19 (13.88) | 63.67 (33.66) |
| Knowledge-Implausible | 16.91 (23.17) | 4.46 (9.54) | 7.76 (16.85) | 70.87 (30.73) |
| Knowledge-Plausible | 24.27 (24.27) | 4.08 (8.42) | 4.23 (11.64) | 67.42 (30.24) |
| **Experiment 5** |  |  |  |  |
| Ignorance | 20.98 (22.37) | 7.75 (12.58) | 5.79 (9.13) | 65.48 (33.74) |
| Knowledge-Implausible | 22.29 (22.56) | 5.71 (8.68) | 8.64 (17.58) | 63.36 (32.06) |
| Knowledge-Plausible | 26.95 (21.59) | 7.51 (12.26) | 7.05 (11.21) | 58.49 (28.19) |
| **Experiment 6** |  |  |  |  |
| Ignorance | 28.18 (23.51) | 6.01 (7.63) | 5.79 (7.69) | 60.03 (28.07) |
| Knowledge-Implausible | 22.05 (21.47) | 4.14 (6.97) | 4.53 (9.55) | 69.27 (27.77) |
| Knowledge-Plausible | 30.86 (25.25) | 2.68 (4.29) | 3.42 (8.7) | 63.04 (26.64) |
| **Experiment 7** |  |  |  |  |
| Ignorance | 16.53 (18.28) | 7.01 (12.62) | 6.95 (12.4) | 69.51 (31.6) |
| Knowledge-Implausible | 16.26 (18.9) | 5.22 (10.28) | 7.45 (15.73) | 71.07 (29.4) |
| Knowledge-Plausible | 22.08 (21.86) | 4.48 (8.49) | 5.12 (11.66) | 68.32 (28.07) |
